# 

**Published:** 1856-01-01

**Authors:** 


### THE JOURNAL

OF

### PSYCHOLOGICAL MEDICINE

AND

### MENTAL PATHOLOGY.

S.G.O.
[illegible]

EDITED BY

FORBES WINSLOW, M.D., D.C.L. Oxon.

VOL. IX.

Surgeon Gen'l's Office
LIBRARY.
22053
Washington, D.C.

LONDON:
JOHN CHURCHILL, NEW BURLINGTON STREET.
MDCCCLVI.

W
1
JO 857 N

LONDON:
SAVILL AND EDWARDS, PRINTERS, CHANDOS STREET,
COVENT GARDEN.

*WORKS PREPARING FOR PUBLICATION,*

BY

DR. WINSLOW.

---

One Vol. 8vo,

*On Softening, and other Obscure Diseases of the Brain.*

One Vol. 8vo,

*The Physician: His Vocation.*

---

In No. II. of this Journal will be published,

**Part I. of a Series of Papers on**

*Idiocy, and other Forms of Arrest of Intelligence occurring in Early Life,*

BY

DR. WINSLOW.

New Commissioner in Lunacy.—We are happy to announce that R. W. Skeffington Lutwidge, Esq., Barrister-at-law, is appointed one of her Majesty's Commissioners in Lunacy.

